# Brief Parenteral Nutrition Accelerates Weight Gain, Head Growth Even in Healthy VLBWs

**DOI:** 10.1371/journal.pone.0088392

**Published:** 2014-02-19

**Authors:** Naho Morisaki, Mandy B. Belfort, Marie C. McCormick, Rintaro Mori, Hisashi Noma, Satoshi Kusuda, Masanori Fujimura, the Neonatal Research Network of Japan

**Affiliations:** 1 Department of Health Policy, National Center for Child Health and Development, Setagayaku, Tokyo, Japan; 2 Department of Pediatrics, University of Tokyo, Bunkyoku, Tokyo, Japan; 3 Division of Newborn Medicine, Boston Children's Hospital, Boston, Massachussetts, United States of America; 4 Harvard Medical School, Boston, Massachusetts, United States of America; 5 Department of Neonatology, Beth Israel Deaconess Medical Center, Boston, Massachusetts, United States of America; 6 Society, Health, and Human Development, Harvard School of Public Health, Boston, Massachusetts, United States of America; 7 Department of Data Science, The Institute of Statistical Mathematics, Tachikawa Tokyo, Japan; 8 Department of Neonatology, Maternal and Perinatal Center, Tokyo Women's Medical University, Shinjukuku, Tokyo, Japan; 9 Department of Neonatology, Osaka Medical Center and Research Institute for Maternal and Child Health, Osaka, Osaka, Japan; Indian Institute of Science, India

## Abstract

**Introduction:**

Whether parenteral nutrition benefits growth of very low birth weight (VLBW) preterm infants in the setting of rapid enteral feeding advancement is unclear. Our aim was to examine this issue using data from Japan, where enteral feeding typically advances at a rapid rate.

**Methods:**

We studied 4005 hospitalized VLBW, very preterm (23–32 weeks' gestation) infants who reached full enteral feeding (100 ml/kg/day) by day 14, from 75 institutions in the Neonatal Research Network Japan (2003–2007). Main outcomes were weight gain, head growth, and extra-uterine growth restriction (EUGR, measurement <10^th^ percentile for postmenstrual age) at discharge.

**Results:**

40% of infants received parenteral nutrition. Adjusting for maternal, infant, and institutional characteristics, infants who received parenteral nutrition had greater weight gain [0.09 standard deviation (SD), 95% CI: 0.02, 0.16] and head growth (0.16 SD, 95% CI: 0.05, 0.28); lower odds of EUGR by head circumference (OR 0.66, 95% CI: 0.49, 0.88). No statistically significant difference was seen in the proportion of infants with EUGR at discharge. SGA infants and infants who took more than a week until full feeding had larger estimates.

**Discussion:**

Even in infants who are able to establish enteral nutrition within 2 weeks, deprivation of parenteral nutrition in the first weeks of life could lead to under nutrition, but infants who reached full feeding within one week benefit least. It is important to predict which infants are likely or not likely to advance on enteral feedings within a week and balance enteral and parenteral nutrition for these infants.

## Introduction

Guidelines for nutritional support of very low birth weight (VLBW, <1500 grams) and very preterm (<32 completed weeks' gestation) infants often emphasize parenteral nutrition as the main source of nutrition for the first weeks of life [1], [2]. However, some infants are able to advance enteral nutrition quickly, particularly infants fed breast milk [3]–[6]. Additionally, providing parenteral nutrition has risks, such as infection, thrombosis, and other complications associated with central venous access [7], and cholestasis [8], and also costs more than feeding enterally. Given the important benefits of early enteral feeding [9], as well as the risks and cost inherent to providing parenteral nutrition, it is important to quantify the benefit of parenteral nutrition in the context of rapid advancement of enteral nutrition. In other words, it is important to clarify the extent to which infants who reach full enteral nutrition at a rapid rate also benefit from parenteral nutrition. This information would inform guidelines about the routine vs. selective parental nutrition use for VLBW preterm infants, particularly those infants expected to achieve full enteral feeding at a rapid rate.

The aim of this study was to quantify the growth benefit of parenteral nutrition in VLBW preterm infants who are able to advance enteral feedings rapidly. Our analysis capitalizes on the Japanese experience in which advancement of enteral feeding in the neonatal intensive care unit (NICU) is typically more rapid than in other countries, possibly due to the high rate of breast milk feeding for preterm infants and the low background necrotizing enterocolitis (NEC) rate [10], [11]. Historically the rate of NEC is below 1% in VLBW infants in the Japanese Neonatal Research Network, as compared with 7–10% in the U.S. or other populations [11]–[13]. We hypothesized that despite routine rapid enteral feeding advancement, parenteral nutrition would nonetheless be beneficial to growth.

## Methods

### Design, Setting, and Participants

We used data from the Neonatal Research Network of Japan (NRN), a multi-center registry of VLBW infants cared for in 75 participating level III perinatal centers in Japan and funded by a grant from the Ministry of Health, Labor and Welfare in 2004. All registered hospitals provided individual patient data including obstetric, delivery, in-hospital care, and follow-up data at age 1.5 and 3 years to the central committee. The central committee identified potential data errors and requested that individual institutions correct data by going back to medical charts when needed. A description of the NRN cohort including morbidities, mortalities, and outcomes at age 1.5 and 3 years, has been reported previously [14]–[16].

**Figure 1:**
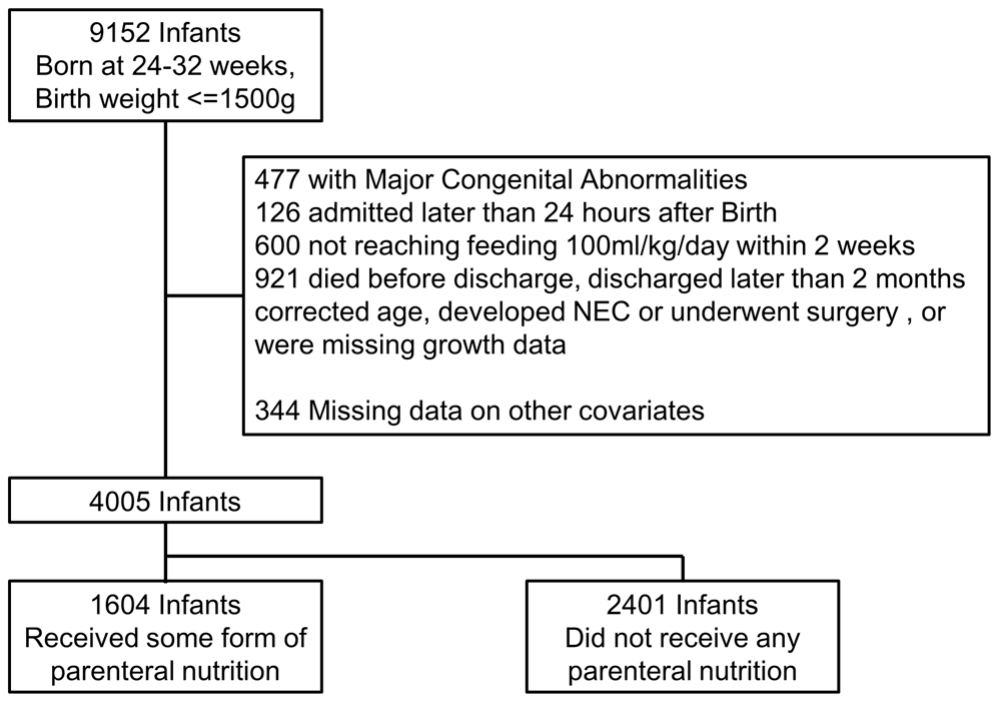
Population Flow Chart.

Figure 1 outlines the population used for this analysis. The NRN cohort included 16001 VLBW infants who were hospitalized in 75level III NICU's in Japan from January 2003 to December 2007. Of these, 9152 were born at 24–32 weeks of gestation. We excluded infants with major congenital anomalies (n = 477) and infants admitted to the NICU more than 24 hours after birth (n = 126). In the remaining 8549 infants, 61% of the infants reached 100 ml/kg/day full feeding within 14 days, and 16% achieved this within 7 days. As our interest was mainly in the effect of parenteral nutrition on growth in infants who reached full enteral nutrition fairly rapidly, we restricted our analysis to infants who reached full feeding within 14 days (n = 5270), of whom 41% (n = 1784) had received parenteral nutrition during admission. We further excluded infants who died before discharge (n = 39), infants discharged after 48 weeks corrected age (n = 348); infants who developed NEC (n = 13) or underwent surgery (n = 385); and infants missing growth data (n = 108). We also excluded 344 subjects due to missing covariate data. Thus our total sample size for analysis was 4005.

### Definition of Diseases and Outcomes

Our main exposure was any use of parenteral nutrition during the hospital stay, which the hospitals provided as yes, or no for each subject. No subject was missing this information.

Our main outcome was extra-uterine weight gain and head growth, which we defined as the change in SD score of each measurement from birth to discharge. These SD scores were calculated using sex, parity, and gestational length (by day) specific growth references for birth weight [17] and head circumference, obtained from vaginal deliveries during 2003–2005 in Japan. We also classified infants as being small for gestational age (SGA) or having extra-uterine growth restriction (EUGR), both proxies for intra-uterine and extra-uterine growth. SGA was defined as birth weight being under the 10^th^ percentile of the reference; EUGR was defined as weight and head circumference at less than the 10^th^ percentile at a given postmenstrual age, as compared with infants born at the same gestational age in the reference.

As growth can be affected by different obstetric and pediatric characteristics as well as the wellbeing of the infant, we considered as covariates the following: maternal age, parity, gestational diabetes (GDM), pregnancy induced hypertension (PIH), use of antenatal steroid, multiplicity, route of delivery, gestational length, sex, SD scores of weight and head circumference at birth, Apgar score at 5 minutes, use of mechanical ventilation, diagnosis and stage of intra-ventricular hemorrhage (IVH), diagnosis of bronchopulmonary dysplasia (BPD), periventricular hemorrhage (PVL), and days taken to reach full enteral feeding.

In all analyses we categorized these variables as follows: maternal age (14–20, 20–34, 35–50 years), parity (0, 1, 2 and above) multiplicity (singleton, twin, triplets or more), route of delivery (cesarean or vaginal), Apgar score at 5 minutes (0–4, 5–10), use of ventilation (no ventilation, ventilation for under 7 days, ventilation over 7 days), IVH (no IVH, grade 1–2 IVH, grade 3–4 IVH), and each completed week of gestation at birth (24 to 32 weeks).

### Statistical Analysis

First, we compared maternal and infant characteristics between infants who received parenteral nutrition (n = 1604) and those who did not (n = 2401). Next, as extra-uterine growth is affected by both gestational age and intrauterine growth, we stratified our sample by each week of gestation and intrauterine growth status (SGA or not) and examined each of the following across strata as well as by use of parenteral nutrition or not: change in SD scores of weight and head circumference at admission and discharge, and length of hospitalization in days.

To account for potential confounding by maternal and infant characteristics, and to account for clustering within institutions, we performed generalized linear mixed models (logistic regression with random intercepts) to estimate the effect of parenteral nutrition on our outcomes of interest (weight gain, head growth, odds of EUGR, and days of admission). For selection of confounders we used parity, GDM, PIH, use of antenatal steroid, multiplicity, gestational length, sex, Apgar score at 5 minutes, IVH stage, use of mechanical ventilation, day of reaching full enteral feeding, as well as birth measurements, due to their clinical relevance and usage in previous papers [5], [18], [19].

We performed all analyses using SAS version 9.3 (SAS Institute, Cary, NC).

### Ethics Statement

All information about the infants was collected anonymously, and the stored data were unlinked from individual data. Written informed consent was obtained from the parents or guardians on behalf of each child enrolled in this study before receiving any data. The protocol of this study was approved by the Central Internal Review Board at Tokyo Women's Medical University, where all data were collected and stored. The database was registered as UMIN000006961.

## Results

**Figure 2:**
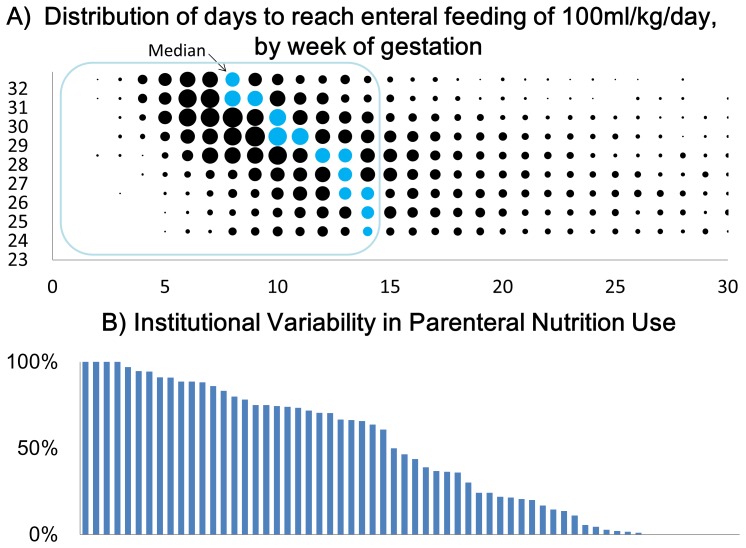
Comparison of nutritional practices in 75 institutions in Japan. A) Distribution of days to reach 100mlperkgperday of milk in 8,549 very low birth weight infants (23–32 weeks). B) Variability in usage of parenteral nutrition in very low birth weight infants (23–32 weeks) who reached full enteral feeding within 2 weeks.

In **Figure 2a** we show the distribution of days until enteral feeding 100 ml/kg/day (full feeding) within 8549 infants without major congenital anomalies and were admitted within 24 hours after birth, by each week of gestation. The distribution was skewed to the right, with 61% of the infants reaching full feeding within 14 days, and 16% achieving this within 7 days. Time to reach full enteral feeding was greater for infants of lower gestational age, but over 50% of infants born in each gestational week reached full feeding within 14 days. This was as expected from the customs in Japanese NICUs which prefer to initiate and increase enteral feeding as soon as possible.

In **Figure 2b**, we show the wide inter-institutional variation in usage of parenteral nutrition.

In **Table 1** we show maternal and infant characteristics for the 4005 infants who reached full enteral feeding by day 14, grouped by whether they received parenteral nutrition.

**Table 1:** Maternal and infant characteristics of 4,005 very low birth weight infants of 24–32 weeks' of gestation who reached full enteral feeding within 2 weeks.

		Infants who did not receive parenteral nutrition (n = 2401)	Infants who received parenteral nutrition (n = 1604)
		Mean (SD) or percentage	
Maternal Characteristics			
Maternal age**		30.6 (5.1)	31.2(5.1)
Number of previous deliveries		0.7(0.8)	0.6(0.8)
Number of fetuses		1.3(0.6)	1.3(0.6)
Gestational diabetes(%)		1.6%	1.8%
Pregnancy induced hypertension(%)		19.3%	19.5%
Use of antenatal steroids(%)**		42.2%	48.8%
Cesarean section (%)		77.3%	78.0%
Infant Characteristics			
Gestational length (weeks)**		29.7 (2.0)	28.7 (2.2)
Length of stay (days)**		75.6 (25.2)	84.2 (26.8)
Apgar score at 5 minutes**		8.1 (1.4)	7.7 (1.7)
Days to reach 100 ml per kg per day enteral feeding**		8.9 (2.7)	10.3 (2.6)
Birth Weight (grams)		1176 (234)	1053 (257)
Weight for gestational age, at birth (SD)		−0.90(1.0)	−0.94 (1.2)
Birth Head Circumference (cms)**		26.6 (2.0)	25.7 (2.2)
Head Circumference for gestational age, at birth (SD)		−0.20 (0.8)	−0.21 (0.8)
Weight at discharge (grams)**		2649 (452)	2713 (497)
Head Circumference at Discharge (cms)		34.2 (1.8)	34.3 (1.8)
Male (%)		49.8%	51.8%
Mechanical ventilation**	No use (%)	24.2%	24.2%
	Less than 1 week (%)	33.4%	33.4%
	More than 1 week (%)	42.4%	42.4%
Intra-Ventricular Hemorrhage**	None(%)	91.0%	91.0%
	Grade 1–2 (%)	7.1%	7.1%
	Grade 3–4 (%)	1.9%	1.9%
PPHN (%)**		1.4%	2.9%
Sepsis (%)**		2.1%	3.8%
BPD (%)**		21.6%	33.4%
PVL (%)		2.8%	3.4%
EUGR by weight (%)		58%	58%
EUGR by head circumference (%)		12%	12%

Full enteral feeding: 100 ml per kg per day of milk.

*: p<0.05.

**: p<0.005.

Infants who received parenteral nutrition were born earlier and were generally sicker, for example they were more likely to have needed mechanical ventilation, were more likely to have BPD, and took longer to reach full enteral feeding. Those receiving parenteral nutrition also had lower weight and smaller head circumference at birth, but showed higher weight and larger head circumference at discharge.

**Figure 3:**
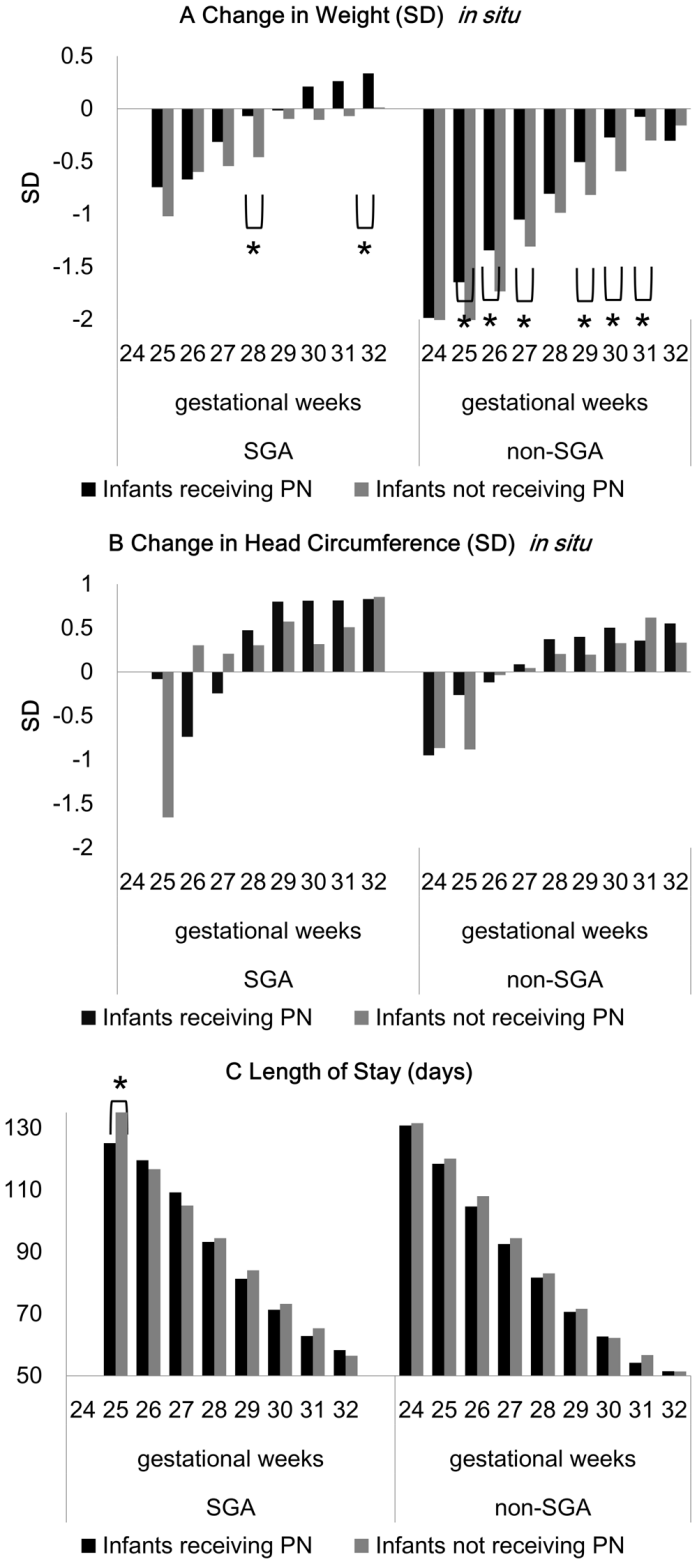
Comparison of infants that received and did not receive parental nutrition, stratified by gestational age and intrauterine growth. A) Change in weight (SD) *in situ*, B) Change in head circumference (SD) *in situ*, and C) Length of stay (days), of 4,005 very low birth weight infants of 24–32 weeks of gestation that reached full enteral feeding within 2 weeks. Figure legends for Figure 3: Full enteral feeding: 100 ml per kg per day of milk. PN: parenteral nutrition. SGA: small for gestational age, defined as birth weight <10^th^ percentile for postmenstrual age.

In **Figure 3** we show the average change in weight and head circumference SD from birth to discharge, as well as length of stay, for infants who received and did not receive parenteral nutrition, stratified by intrauterine growth (SGA and non-SGA) as well as week of gestation. Even after stratification, infants receiving parenteral nutrition showed greater growth in most categories.

**Table 2** shows the estimated effect of administering parenteral nutrition on growth and length of stay, adjusted for maternal and infant characteristics listed in **Table 1**. Infants receiving parenteral nutrition showed greater weight gain and head growth: on average 0.09 (95% confidence interval [CI] 0.02, 0.16) SD greater weight gain and 0.16 (95% CI 0.05, 0.28) SD greater head growth. They also tended to have lower odds of being EUGR by weight (OR 0.85, 95% CI 0.66, 1.08) and head circumference (OR 0.66, 95% CI 0.49, 0.88) at discharge, compared to those not receiving parenteral nutrition. Length of stay was 1.29 (95% CI 0.12, 2.45) days shorter for infants who received parenteral nutrition. There was no significant association of parenteral nutrition with adverse outcomes: BPD (OR: 0.85; 95%CI 0.66–1.08); and PVL (OR: 1.19; 95%CI 0.76–1.87).

**Table 2:** Adjusted effect of parenteral nutrition use on weight, head circumference, and length of NICU stay.

	Difference (95% CI)	p-value
Growth parameters in z-scores		
Weight gain (SD)	0.09 (0.02, 0.16)	0.01
Head growth (SD)	0.16 (0.05, 0.28)	0.004
Length of stay (days)	−1.29 (0.12, 2.45)	0.03

	Odds ratio (95% CI)	p-value
Growth parameters by Extrauterine Growth		
EUGR by weight (OR)	0.85 (0.66, 1.08)	0.18
EUGR by head circumference (OR)	0.66 (0.49, 0.88)	0.005

Analysis of 4,005 very low birth weight infants of 24–32 weeks' gestation who reached full enteral feeding within 2 weeks.

Generalized linear mixed models (logistic regression with random intercepts) used to accounting for clustering within institutions. Adjusted for selected maternal (maternal age, number of previous deliveries, number of fetuses, gestational diabetes, pregnancy induced hypertension, use of antenatal steroids, mode of delivery), and infant (gestational length, sex, birth weight, birth head circumference, Apgar score at 5 minutes, days to reach 100 ml per kg per day enteral feeding, length of stay) characteristics.

Full enteral feeding: 100 ml per kg per day of milk.

EUGR: Extra-uterine growth restriction; defined as weight or head circumference <10^th^ percentile for postmenstrual age.

**Figure 4:**
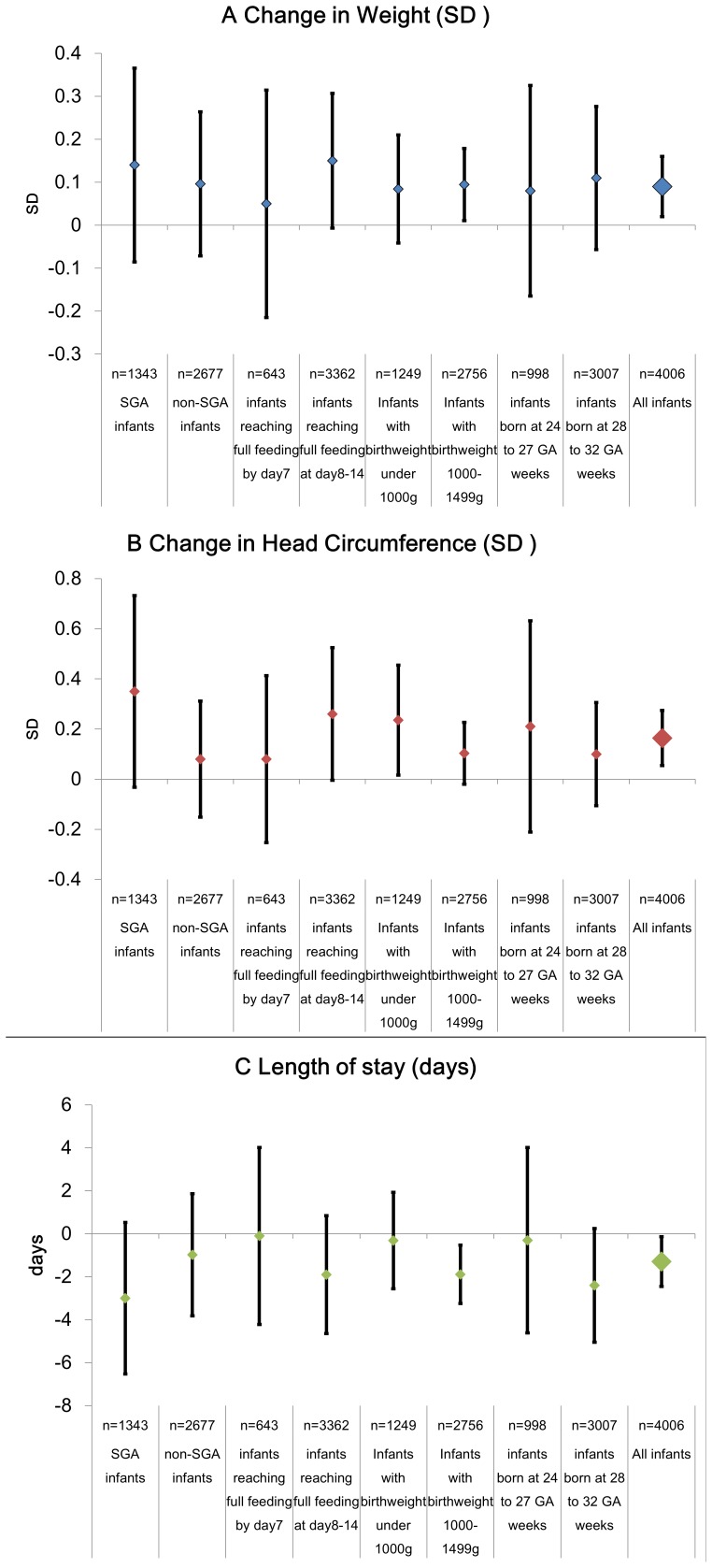
Estimated effect of administering parenteral nutrition. A) Change in weight (SD) *in situ*, B) Change in head circumference (SD) *in situ*, and C) Length of stay (days). Analysis of 4,005 very low birth weight infants of 24–32 weeks of gestation who reached full enteral feeding within 2 weeks. Legends for Figure 4: Generalized linear mixed models (logistic regression with random intercepts) used to accounting for clustering within institutions. Adjusted for selected maternal (maternal age, number of previous deliveries, number of fetuses, gestational diabetes, pregnancy induced hypertension, use of antenatal steroids, mode of delivery), and infant (gestational length, sex, birth weight, birth head circumference, Apgar score at 5 minutes, days to reach 100 ml per kg per day enteral feeding, length of stay) characteristics. Full enteral feeding: 100 ml per kg per day of milk.

Effect modification by weeks of gestation (24–27 weeks or 28–32 weeks), intrauterine growth (SGA or non-SGA), and day to reach full feeding (0–7 days or 8–14 days) were not statistically significant, and though estimates differed slightly for each subgroup, as shown in **Figure 4**, the all effects were in the same direction.

However, point estimates of the effect of parenteral nutrition was largest in SGA infants and infants who took more than one week until full feeding, and smallest in infants who reached full feeding within a week. Point estimates of increase in weight was largest in SGA infants (0.14SD; 95%CI −0.09, 0.37) and infants reaching full feeding after one week (0.08SD; −0.01, 0.31), and smallest in infants who reached full feeding within one week (0.05SD; −0.22, 0.32). Similarly, point estimates of increase in head circumference was largest in SGA infants (0.35SD; −0.03, 0.73) and smallest in non-SGA infants (0.10SD; 0.15, 0.73) and infants reaching full feeding within one week (0.08SD; −0.25, 0.41). Point estimates for effect on length of stay was also largest in SGA infants and infants born at 28 to 32 weeks of gestation, and nearly null in infants who reached full feeding within a week, were born under 1000 g, or were born at 24–27 weeks of gestation.

## Discussion

We observed that among VLBW preterm (24–32 weeks' gestation) infants who reached full enteral feedings within 2 week of birth, those infants provided with parenteral nutrition demonstrated greater weight gain and head growth, and a lower prevalence of EUGR by weight, as compared with those who did not receive parenteral nutrition. To the best of our knowledge, this study is the first to show that parenteral nutrition promotes weight gain even in preterm infants who are able to advance enteral nutrition at a fairly fast rate.

Research to inform optimal nutritional care of the preterm infant is important because inadequate nutrition leads to poor growth extrauterine growth, which in turn has been linked with poor later neurocognitive outcomes [20]–[22]. EUGR is highly prevalent among VLBW and ELBW infants [20], [23] and can be modified by nutritional practices in the NICU [24], [25]

Historically, clinicians have been hesitant to advance enteral feedings at a rapid rate due to concerns about intestinal immaturity and the associated risk of NEC, a serious and often life-threatening complication of preterm birth. [26]. Research focused on providing earlier parenteral nutrition, such as intravenous amino acids, has shown a beneficial impact on growth of VLBW infants [27]. For example, Poindexter reported that early provision of amino acids was associated with significantly better growth at 36 weeks postmenstrual age. Similarly, Maggio reported changes in parenteral nutrition practice improved growth outcomes at discharge. Our results are consistent with both of those studies in supporting the benefit of parenteral nutrition on growth of VLBW infants.

However, in other studies of early parenteral nutrition and growth, enteral feedings were advanced slowly. In the Poindexter study, subjects took on average 32–34 days to reach 110 kcal/kg/day enteral feeding, and were administered parenteral nutrition for on average 32 days [28]. In the Maggio study, subjects took on average 24–27 days to reach 150 ml/kg/day enteral feeding, and were administered parenteral nutrition for 24–27 days [27]. Even the recent studies shown in a meta-analysis by Moyses [9] on the effect of parenteral nutrtion on growth, show that time to full feeds took an average of 15 to 33 days [29]–[33]. In contrast, in our study population, enteral feedings were advanced much more quickly, on average over 8–10 days. To our knowledge, no study prior to ours has evaluated the impact of parenteral nutrition in the setting of more rapid advance of enteral feedings.

Additionally, through subgroup analysis we found that overall, SGA infants and infants reaching full feeding after one week seemed to benefit most from parenteral nutrition in means of growth and shorter stay. Infants who reached full feeding within one week seemed to benefit least. Our findings suggest that it is important to predict which infants are likely or not likely to advance on enteral feedings within a week.

Our study has several limitations. First, we did not have data on the timing or duration of parenteral nutrition. Therefore we had to exclude infants who would be receiving parenteral nutrition due to their complications: infants that had NEC, surgery, or died during hospitalization, and thus we could not set these conditions as outcomes. However, we were most interested in the effects of parenteral nutrition in otherwise healthy preterm infants. Second, although a number of maternal and child characteristics were available, residual confounding may occur by characteristics of infants who received parenteral nutrition that could promote their growth, for instance other differences in nutritional practice. Finally, our study was conducted in Japan where neonatal intensive care practices differ from the U.S. and other countries. However, our setting provides a unique opportunity to examine the role of parenteral nutrition for infants who advance quickly on enteral nutrition, and we believe our findings will be relevant for non-Japanese populations of VLBW infants as well.

In summary, our results support the use of parenteral nutrition to improve weight gain and head growth, even among relatively healthy VLBW infants who reach full enteral feeding within 2 weeks. For infants reaching full enteral feedings in 1 week or less, the benefit was smaller. These findings will be useful for clinicians weighing the risks and benefits of providing parenteral nutrition to very low birth weight infants, particularly those who are expected to advance enteral nutrition rapidly.
