# Correction: Brief Parenteral Nutrition Accelerates Weight Gain, Head Growth Even in Healthy VLBWs

**DOI:** 10.1371/journal.pone.0143984

**Published:** 2015-11-25

**Authors:** Naho Morisaki, Mandy B. Belfort, Marie C. McCormick, Rintaro Mori, Hisashi Noma, Satoshi Kusuda, Masanori Fujimura, Neonatal Research Network of Japan

There is an error in the third-to-last sentence of the “Design, Setting, and Participants” subsection in the Methods. The correct sentence is: We further excluded infants who died before discharge (n = 39), infants discharged after 48 weeks corrected age (n = 348); infants who developed NEC (n = 13) or underwent surgery (n = 385); and infants missing growth data (n = 178).

In Fig 1, the second box of the flow chart contains incorrect information. “600” should be “3279.” Please see the corrected Fig 1 here.

**Fig 1:**
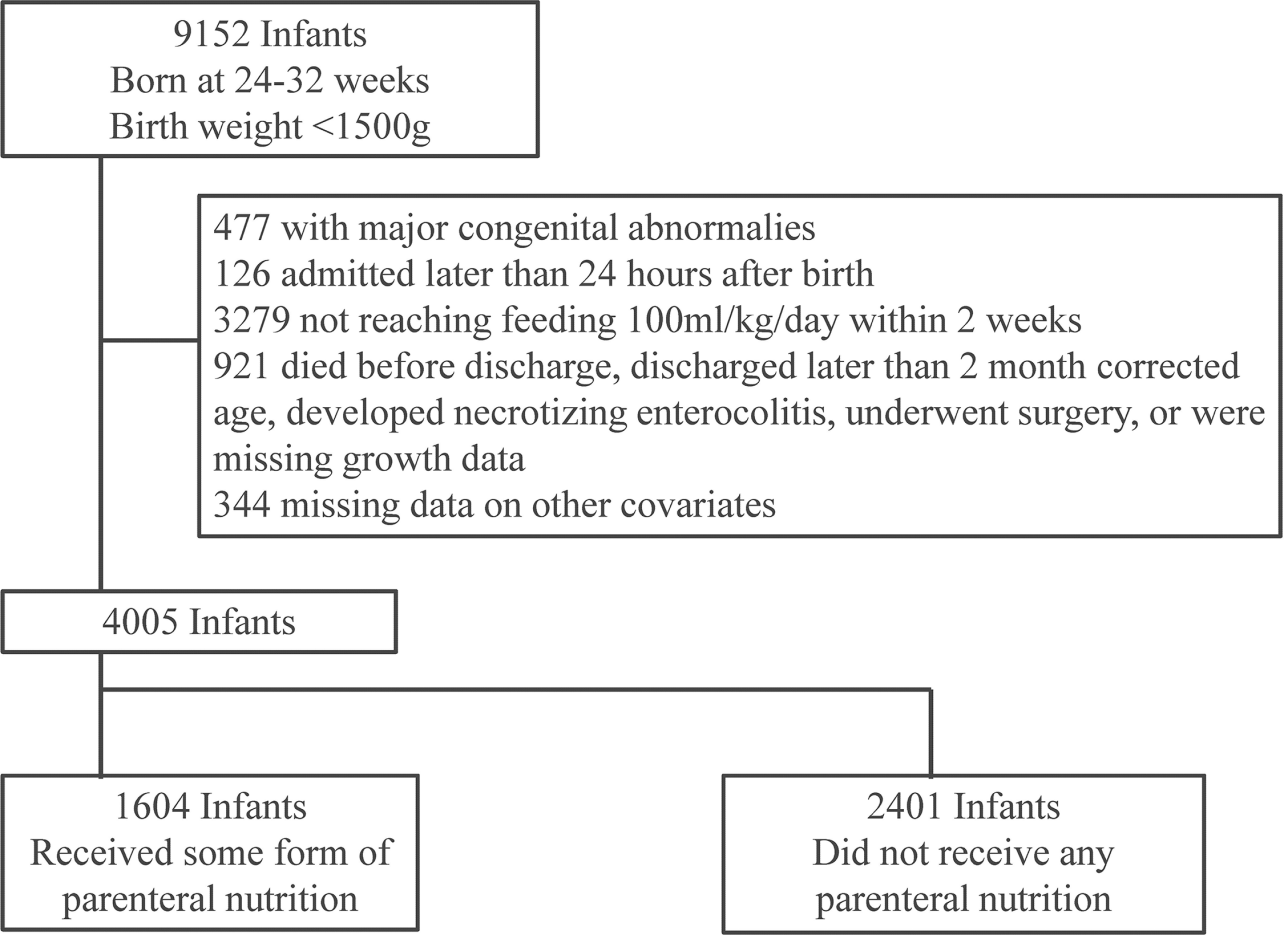
Population Flow Chart

In Fig 2, the axis labels are missing from both panels. Please see the corrected Fig 2 here.

**Fig 2:**
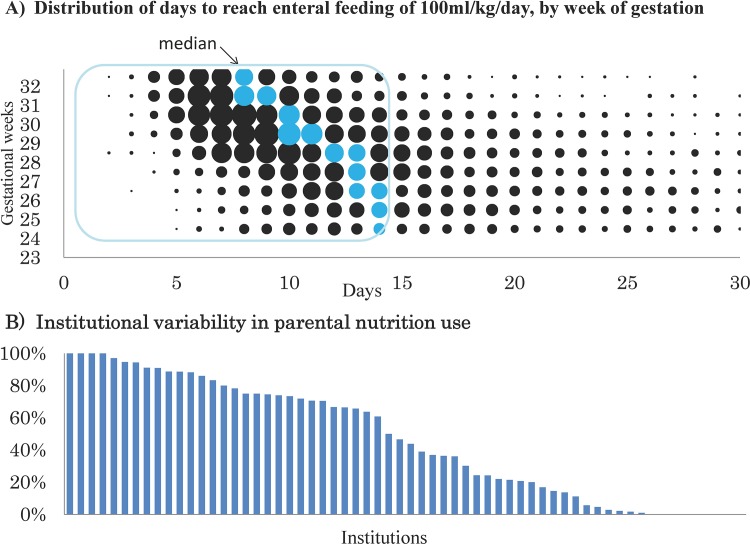
Comparison of nutritional practices in 75 institutions in Japan. A) Distribution of days to reach 100 ml per kg per day of milk in 8,549 very low birth weight infants (23–32 weeks). B) Variability in usage of parenteral nutrition in very low birth weight infants (23–32 weeks) who reached full enteral feeding within 2 weeks.

In Fig 4, there are errors in the axis labels for all three panels. “n = 2677 non-SGA infants” should be “n = 2662 non-SGA infants” and “n = 4006 All infants” should be “n = 4005 All infants.”

**Fig 4:**
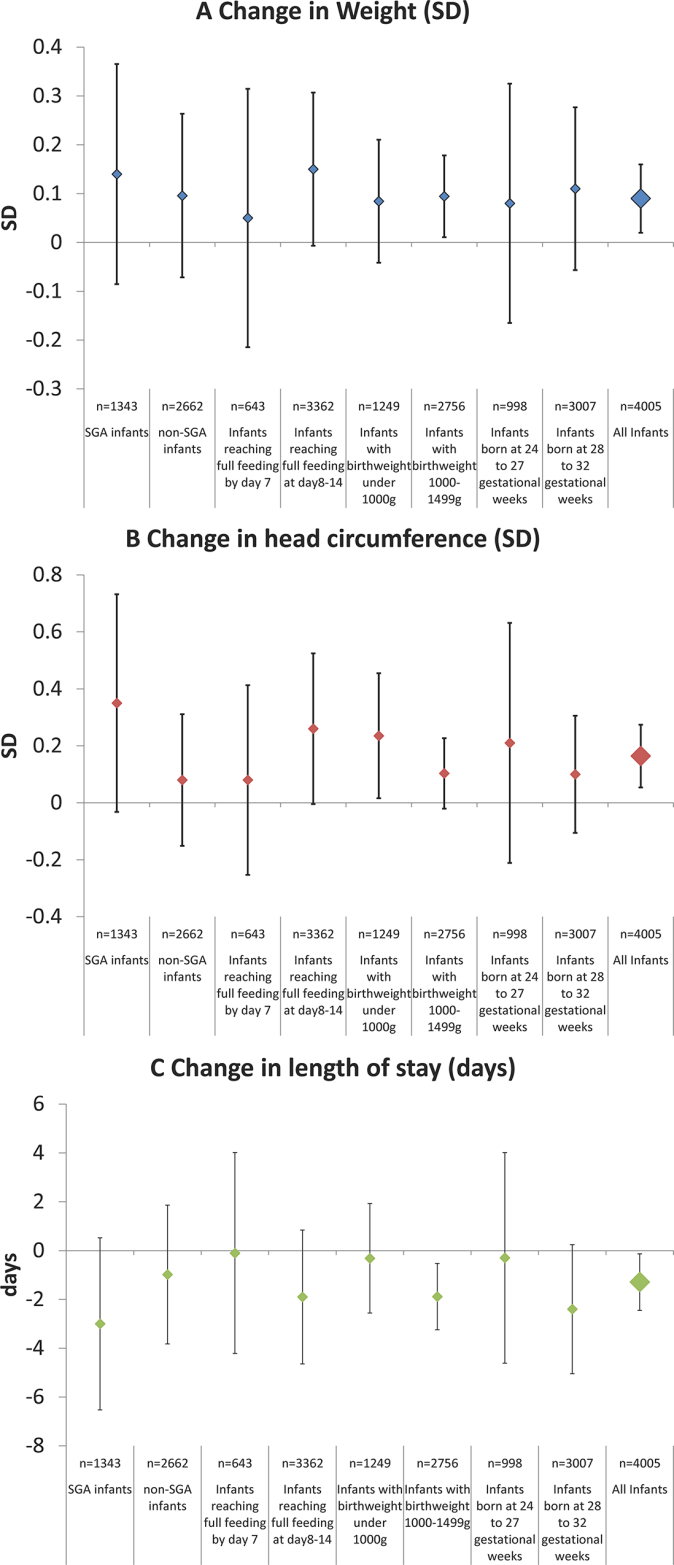
Estimated effect of administering parenteral nutrition. A) Change in weight (SD) in situ, B) Change in head circumference (SD) in situ, and C) Length of stay (days). Analysis of 4,005 very low birth weight infants of 24–32 weeks of gestation who reached full enteral feeding within 2 weeks. Legends for Fig 4: Generalized linear mixed models (logistic regression with random intercepts) used to accounting for clustering within institutions. Adjusted for selected maternal (maternal age, number of previous deliveries, number of fetuses, gestational diabetes, pregnancy induced hypertension, use of antenatal steroids, mode of delivery), and infant (gestational length, sex, birth weight, birth head circumference, Apgar score at 5 minutes, days to reach 100 ml per kg per day enteral feeding, length of stay) characteristics. Full enteral feeding: 100 ml per kg per day of milk.

There are a number of errors in Table 1. Please see the complete, correct Table 1 here.

**Table 1:** Maternal and infant characteristics of 4,005 very low birth weight infants of 24–32 weeks’ of gestation who reached full enteral feeding within 2 weeks.

		Infants who did not receive parenteral nutrition (n = 2401)	Infants who received parenteral nutrition (n = 1604)
		Mean (SD) or percentage	
Maternal Characteristics			
Maternal age **		30.6 (5.1)	31.2 (5.1)
Number of previous deliveries		0.7 (0.8)	0.6 (0.8)
Number of fetuses		1.3 (0.6)	1.3 (0.6)
Gestational diabetes (%)		1.6%	2.1%
Pregnancy induced hypertension (%)		19.7%	20.2%
Use of antenatal steroids (%)		42.9%	49.1%
Cesarean section (%)		78.0%	78.0%
Infant Characteristics			
Gestational length (weeks) **		29.7 (2.0)	28.7 (2.2)
Length of stay (days) **		75.6 (25.2)	84.2 (27.0)
Apgar score at 5 minutes **		8.1 (1.4)	7.7 (1.7)
Days to reach 100ml per kg per day enteral feeding **		8.9 (2.7)	10.3 (2.5)
Birth Weight (grams)		1172 (235)	1049 (258)
Weight for gestational age, at birth (SD)		-0.90 (1.0)	-0.94 (1.2)
Birth Head Circumference (cms) **		26.6(2.0)	25.7 (2.2)
Weight at discharge (grams) **		2645 (447)	2707 (495)
Head Circumference at discharge (cms)		34.2 (1.8)	34.3 (1.7)
Male (%)		49.7%	51.8%
Mechanical ventilation **	none (%)	41.7%	24.9%
	Less than 1 week (%)	35.6%	32.7%
	More than 1 week (%)	22.7%	42.4%
Intra-Ventricular Hemorrhage **	none (%)	94.5%	90.7%
	Grade 1–2 (%)	4.1%	7.4%
	Grade 3–4 (%)	1.0%	1.9%
Persistent Pulmonary Hypertension of the Newborn(%) **		1.4%	2.9%
Sepsis(%) **		2.2%	3.7%
Bronchopulmonary dysplasia (%) **		21.7%	32.9%
Periventricular Hemorrhage (%)		2.8%	3.5%
Extra-uterine growth restriction by weight (%)		58.1%	57.9%
Extra-uterine growth restriction by head circumference (%)		11.5%	11.5%

Full enteral feeding: 100 ml per kg per day of milk.

*: p,0.05.

**: p,0.005.
